# Nucleosome distortion as a possible mechanism of transcription activation domain function

**DOI:** 10.1186/s13072-016-0092-2

**Published:** 2016-09-20

**Authors:** Tamara Y. Erkina, Alexandre M. Erkine

**Affiliations:** Department of Pharmaceutical Sciences, College of Pharmacy and Health Sciences, Butler University, Indianapolis, IN 46208 USA

**Keywords:** Transcriptional activation domain, Transcription regulation, Chromatin, Chromatin remodeling, Intrinsically disordered region

## Abstract

After more than three decades since the discovery of transcription activation domains (ADs) in gene-specific activators, the mechanism of their function remains enigmatic. The widely accepted model of direct recruitment by ADs of co-activators and basal transcriptional machinery components, however, is not always compatible with the short size yet very high degree of sequence randomness and intrinsic structural disorder of natural and synthetic ADs. In this review, we formulate the basis for an alternative and complementary model, whereby sequence randomness and intrinsic structural disorder of ADs are necessary for transient distorting interactions with promoter nucleosomes, triggering promoter nucleosome translocation and subsequently gene activation.

## Background

Transcriptional activators are the main triggers of gene activation, determining not only the specific selection of genes transcribed in response to distinct stimuli, but also the level of transcription and its modulation [1–4]. A typical activator molecule consists of at least two critical parts: a DNA-binding domain (DBD), which determines specificity of binding to a distinct promoter DNA sequence, and a transcription activation domain (AD), which determines initiation of transcription and its level. Additional domains responsible for oligomerization, cellular localization, degradation, and other functions vary greatly between activators. DNA-binding domains are well characterized both by specificity of promoter sequence recognition and by distinct structural features [5–7]; ADs, however, despite being discovered more than three decades ago, remain largely enigmatic [3, 8]. Although the broadly accepted mechanism of ADs’ action involves direct physical recruitment of a wide variety of enzymatic activities and protein complexes associated with chromatin remodeling and transcription initiation, the order of recruitment events, structural determinants, and specific amino acid sequences involved in recruitment events are often not clear and/or are contradictory.

The growing number of human diseases associated with the malfunction of gene expression argues for the development of new drugs affecting specific transcriptional activators or functionally substituting them [9–11]. Technical capabilities for small-molecule artificial activators have been demonstrated by a chemical synthesis of both functional DBDs [7, 9, 12] and ADs [13, 14]. Nevertheless, the development of artificial activators with specific transcription activation potential is greatly hampered by deficiencies in our understanding of mechanisms of ADs’ function, which have been documented by different researchers over the past two decades [3, 8, 15, 16]. Thus, elucidation of ADs’ functional mechanism is critically important for different areas of molecular biology and medicine.

## Review

### ADs’ properties and characteristics

#### Interchangeability of ADs

One of the remarkable properties of ADs is the general functional interchangeability among gene-specific activators. Testing all 6000 ORFs of the yeast genome for functionality as activators in the context of Gal4 DBD, and *GAL1*-*HIS3* and *GAL1*-*LacZ* reporters revealed that 92 known yeast activators can work in this new context, with 32 showing strong activation potential [17]. This functional conservation and ease of functional interchangeability are not restricted to a particular intracellular environment. Moreover, such interchangeability is also easily observed for transcriptional activators belonging to such widely diverse phyla as mammals, fungi, and plants. The most vivid examples are such ADs as VP16 (derived from herpes simplex virus protein Vmw65), yeast Gal4 AD (used in variety of two-hybrid screening systems), and human p53 AD [1, 18–22].

#### Absence of structural and sequence conservation among ADs

Based on the ease of interchangeability, it is logical to expect some similarities in structure and/or sequence composition. Contrary to these expectations, comparison of the amino acid sequences of known ADs does not reveal any amino acid consensus or specific structural motif. In fact, the majority of known natural ADs have no distinct structure and are characterized by a high level of intrinsic disorder [23, 24]. Despite being so widely diverse, ADs can be divided with some approximation into different categories based on their composition: acidic, glutamine-rich, and proline-rich [25], with the former type being the most abundantly represented in higher eukaryotes and nearly the only type found in yeast.

#### ADs amino acid composition

Over the years, there have been several attempts to gain insight into ADs’ composition and other characteristics using a yeast one-hybrid fusion protein system, whereby random sequences were screened for functionality as ADs while connected to a variety of DBDs using a viability test based on a single reporter. The pioneering screen was done by Ma and Ptashne in 1987, testing randomly fragmented *E. coli* genomic DNA fragments fused to the Gal4 DBD [26]. The result of this screen was the realization that approximately 1 % of all library sequences possess *bona fide* AD potential and that these sequences are significantly enriched with acidic residues. Later it was shown that hydrophobic amino acids are equally as important [27–30]. A similarly high percentage of random sequences functioning as ADs and also having an excess of hydrophobic and acidic residues was identified in a completely different genetic context using HSF C-terminal AD substitution by random yeast genomic DNA fragments [31] and in a screen using a completely artificial fusion library of LexA DBD connected to 45 nucleotides of randomized sequence [32]. The LexA-based screen also identified a small number of active sequences containing a total positive charge.

It is paradoxical that despite the easy functional interchangeability among ADs, even those belonging to transcriptional activators from different phyla, they share no clear consensus sequence, and the only compositional similarity between different interchangeable ADs is an excess of hydrophobic and acidic amino acid residues. Even for this loose rule, there are known exceptions, with some ADs containing a positive total charge or at least an absence of acidic residues [32]. Functionality of polyglutamic acid or polyproline stretches has also been demonstrated [33]. Average frequency of functional AD sequences equal to ~1 % in randomized pools of DNA [26–28, 32] suggests astronomical number of functional AD sequences, for instance equal to 1 % of 4^45^ for a stretch of 45 nucleotides. The high variability of AD sequence compositions, in combination with very high frequency of functional AD sequences isolated from random pools [26–28, 32], suggests low specificity of interactions with potential targets.

#### AD sequence length

The lengths of ADs are another important characteristic. Natural AD length varies over several dozen amino acid intervals. Surprisingly, the above-mentioned screens of randomized sequences revealed that ADs with potency comparable to natural sequences can be as short as 12, 11, nine, or even eight amino acids [26, 27]. In fact, natural AD sequences can generally be reduced in size to these functional minimums without significant loss of potency, although for some ADs it was shown that potency is proportionate to length [34]. Increasing AD length by fusing several AD modules in tandem increases the activity in an additive manner in some cases [35]. Demonstration of short peptides effectively functioning as ADs led to the formulation of a nine-amino-acid pattern prediction algorithm [21]. However, it has been shown that the results of applying this algorithm to well-characterized Gcn4 or Gal4 sequences do not match the prediction [15].

#### Non-natural ADs

The minimal size of functional ADs is not limited to eight amino acids, as it has been shown that the AD peptides can be substituted with non-natural compounds or other molecular sequences that are much smaller [13]. For instance, isoxazolidine (MW 290) can function in vitro as an AD while chemically connected to LexA DBD, acting with the same potency as the 14-amino-acid minimal module of VP16 natural AD (MW 1674) [13]. Several other similar compounds have also been demonstrated to function as ADs both in vivo and in vitro, displaying functionality of compounds that are comparable in MW to two–three amino acids [13, 14, 36]. Importantly, these compounds have negatively charged and aromatic extremities. Similarly puzzling are demonstrations that natural ADs can be replaced in vivo, with retention of functionality, by certain RNA sequences [37] or by the VP16 AD peptide chemically synthesized from non-natural D-amino acids mimicking the natural sequence [38].

#### Intrinsically disordered nature of ADs and structural adaptation upon target binding

Based on conservation of function between different AD sequences, it is logical to expect a conservation of specific structural motifs, as was clearly shown for DBDs [5, 6]. Contrary to these expectations, ADs do not have specific structural motifs and are known to be a clear example of intrinsically disordered protein domains, which is the subject of a new and rapidly developing research field [39–41]. Beginning with their discovery, AD sequences were believed to have an amphipathic helical structure [1, 42]. Indeed, some structural studies of ADs in complex with proteins of the transcriptional machinery [43–45] have demonstrated ADs’ adaptation of the amphipathic helical structure in accordance with the “induced fit” model [46, 47]. This structural motif, although shown only in a limited number (˂15) of structural studies and only for AD–protein complexes [8], remains the only loosely defined structural feature of ADs other than the intrinsically disordered character.

In sum, despite being easily interchangeable, with conservation of function between activators belonging even to different biological phyla, as well as being easily isolatable in large numbers from random pools of sequences, ADs do not share any consensus sequence and/or structural motif, at least in the absence of their interaction partners. The size of ADs is also highly variable, ranging from several dozen to as few as eight amino acid modules. Even the chemical nature is elusive, varying from different amino acids of L or D chirality working almost equally well, to certain RNA sequences, and to relatively small (MW 290) organic compounds. The paradox of conservation of function without conservation of sequence and/or structure shows the deficiency of our understanding of ADs and the necessity for further investigation, perhaps fundamentally revising research strategies and mechanistic concepts.

The commonalities arising from the investigation of a large number of natural and synthetic ADs are limited to the combination of acidity and hydrophobicity, and the intrinsically disordered nature of identified peptides. A large fraction of functional amino acid sequences (up to 1 %) isolated from randomized pools suggests by itself that although some specificity is required, potentially interacting targets in vivo might be numerous, and interactions are likely of low specificity.

### Mechanism of AD function and overview of AD-interacting targets in historical perspective

Since their discovery nearly three decades ago, the mechanism of ADs’ function was proposed to be a direct physical recruitment of transcription-related activities to gene promoters [1, 26, 48]. One of the initial and still standing hypotheses is that the direct interacting target of ADs is TATA box-binding protein (TBP) [49–53]. Subsequent experimental works showed that, in addition, other components of basal transcriptional machinery, such as TFIIB [54, 55], TFIIH [56, 57], TFIIA [58, 59], or RNA polymerase II itself [60], are likely targets of ADs. However, attempts to reconstitute this mechanistic model in vitro with purified components did not produce positive results [8], although this might be due to inadequate in vitro conditions.

A subsequent shift of attention in the transcription field to TBP-associated factors (TAFs) resulted in the proposition that the obligatory interacting targets of ADs are TAFs [61]. However, inactivation or depletion of individual yeast TAFs, including the core TAFII that interacts with TBP, either did not compromise transcriptional activation or only affected it mildly [62, 63], and the affected genes were primarily cell cycle-specific genes.

An alternative school of thought [64, 65] that still stands [15, 66] is that the direct interacting targets of ADs are components of the large (at least 31-subunit) Mediator complex that may serve as a bridge between activators and RNA polymerase [67]. With the use of sophisticated biochemical and genetic experiments, several subunits have been shown to be likely interacting targets of ADs: Med17 (Srb4), Srb10, Med15 (Gal11), Med2, and Med 25 [15, 66, 68–72]. The list is far from being exhaustive, especially if fungi, metazoan, and plant variants of Mediator complex are considered [66, 73–76]. Somewhat contradictory to the concept of direct recruitment of Mediator complex by ADs was the demonstration of activator-independent function of Mediator in the activation of transcription [77]. These data again underscore the multiplicity of ADs’ potential targets and recruitment steps.

Explosive development in the 1990s and early 2000s of the field of chromatin-remodeling and histone-modifying activities, in combination with the demonstration of how critical some of these activities are for the initiation of transcription [78–81], led to the identification of multiple AD-interacting targets among these protein complexes [82–85]. Convincing evidence obtained using both in vivo and in vitro approaches demonstrated Gal4 and VP16 AD interactions with the SAGA complex via subunit Tra1 [86–88], which is also an integral part of the NuA4 histone-modifying complex. Later, Tra1 was utilized as a bait for the creation of a screening system for AD sequences [89]. Curiously, interactions of ADs with SAGA were also demonstrated via other subunits such as Ada2 and Taf17 (current nomenclature TAF9) [90–93]. The same ADs were shown to interact with the Swi1 and Snf5 subunits of the Swi/Snf chromatin-remodeling complex [93–95]. Interestingly, the ADs’ interactions with the Snf2 subunit occurred within two distinct regions [95]. In higher eukaryotes, interaction of ADs was demonstrated with CBP, a multifunctional protein affecting not only histones but also components of transcriptional machinery [96–98]. The list of AD-interacting targets described above is far from being complete and illustrates huge variations of possible targets and their combinations even within the chromatin-remodeling and histone-modifying group of enzymatic activities.

In an attempt to systematize and logically connect the recruitment events involved in chromatin remodeling, the yeast *HO* promoter was subjected to consecutive chromatin immunoprecipitation experiments. The results were indicative of the ordered recruitment of activities. Chromatin remodeling by the SWI/SNF complex was followed by recruitment of SAGA, which in turn facilitated Mediator and Pol II recruitment [99, 100]. But even within this model, the specifics of interactions are lacking, while physical and biochemical reasons for the specific order of recruitment events are not clear. The sequence of the recruitment events for the majority of other gene promoters has not been determined.

Perhaps the culmination of this line of investigation, demonstrating a wide range of possible mechanisms and synergistic interactions on long- and short-range scales, is a recent attempt to investigate the functionality of 223 chimeric activators created by fusion of individual subunits of known chromatin regulators to zinc finger DNA-binding proteins [2]. Although it did not reveal a single specific mechanism or order of recruitment events, this study demonstrated an enormous scale of possible recruitment combinations. This immense complexity demands clarification and mechanistic insight.

In an initial attempt to unite this multiplicity of possible interacting targets in a uniform mechanism, the acidic ADs were proposed to function as “acidic blobs” recruiting numerous protein complexes by simple electrostatic interactions [101, 102]. This view later was significantly revised, and, according to a new model, interactions of ADs with multiple targets are bi-phasic, with the initial binding phase being fast and nonspecific, based on the electrostatic interactions, and the following phase being slower, entropy-driven, and involving activator–target-induced fit [103]. This model is based on in vitro surface plasmon resonance studies and was tested for several possible targets, including TBP, Swi1, and Snf5.

More recent investigation of GCN4–Mediator interactions [15], based on both in vivo and in vitro approaches and involving extensive mutagenesis, led to postulation of a model based on “fuzzy” interactions. This study also confirmed that AD–target interactions do not depend on any one residue within the GCN4 AD and likely are based on multiple potentially redundant contacts. Confirming the challenges related to investigation of ADs, this study once again revealed that after three decades of intensive investigations, we are still not entirely clear on understanding the exact mechanism of ADs’ function.

### Specificity challenge: recruitment of large numbers of potential targets via low-specificity interactions

Summarizing ADs’ properties, interacting targets, and the mechanism of AD function, it is evident that despite enormous efforts over a long period of time, there is no clear mechanistic understanding of ADs’ function. Although each specific AD performs essentially the same function, namely facilitating chromatin remodeling in broad sense and assembly of the transcription initiation complex, ADs do not have a consensus sequence. That is true even for synthetic ADs selected for functioning within the context of the same activator and the same reporter gene(s) [26, 31, 32]. The chemical nature of ADs can vary from amino acid sequences to specific RNA sequences, and even to specific non-natural chemical compounds. Structurally, AD sequences are largely disordered, with some indications of more stable structure adaptation upon interaction with a target. The spectrum of potential targets is vast, and the mechanism of these interactions, as well as the nature of prioritizing these interactions, is not clear. Utilizing the current and commonly accepted direct-recruitment model for AD function leads to the formulation of a number of “hard to explain” questions [3, 8, 15, 16]:

1. Considering that ADs interact with targets utilizing nonspecific, low-affinity “fuzzy” interactions with very few contact points (two–three critical amino acids within 10–12 amino acid blocks or even relatively simple compounds [13, 21]), it is not clear how or whether a specific AD sorts through and chooses the right interacting target among hundreds of potential targets. This is even more puzzling considering, on the one hand, the low abundance of most of the possible targets in the cell nucleus, and on the other, the competition of possible interacting targets with each other, possibly making each individual interaction either insignificant or functionally disruptive.
2. If the best target exists for a specific activator in the context of a specific promoter (as it was suggested for GCN4-Gal11 or CBP-TBP pairs), why does the experimental selection of functional ADs from a pool of random sequences or the evolutionary selection not yield a specific AD consensus sequence? Or similarly, why is there a lack of sequence conservation among ADs?
3. What determines the sequence of recruitment events of seemingly distinct enzymatic complexes to the gene promoter, and how are ADs involved in this process?
4. The structural analysis of AD sequences certainly reveals the absence of a clear motif. In fact, ADs comprise one of the best examples of intrinsically disordered protein domains. Why is the structural disorder apparently so important for ADs’ function?
5. Why does the same AD retain functionality and often the degree of activation potential if transferred within evolutionarily very distant contexts of mammalian, plant, and fungi cells [1, 18–22]?

One can invoke the direct-recruitment model to argue that the high level of randomness of sequence and structure is exactly necessary for the recruitment of multiple targets from a large pool, but this does not help with resolving the first three questions. Challenges regarding understanding the mechanism of ADs’ function have been stated on multiple occasions [3, 8, 15, 16]. The long-standing questions and absence of clear mechanistic explanation for ADs’ function suggest the necessity for a revision or at least for flexibility in consideration of current mechanistic concepts of ADs’ function.

### AD–nucleosome interactions as an alternative addition to direct-recruitment model

**Fig. 1:**
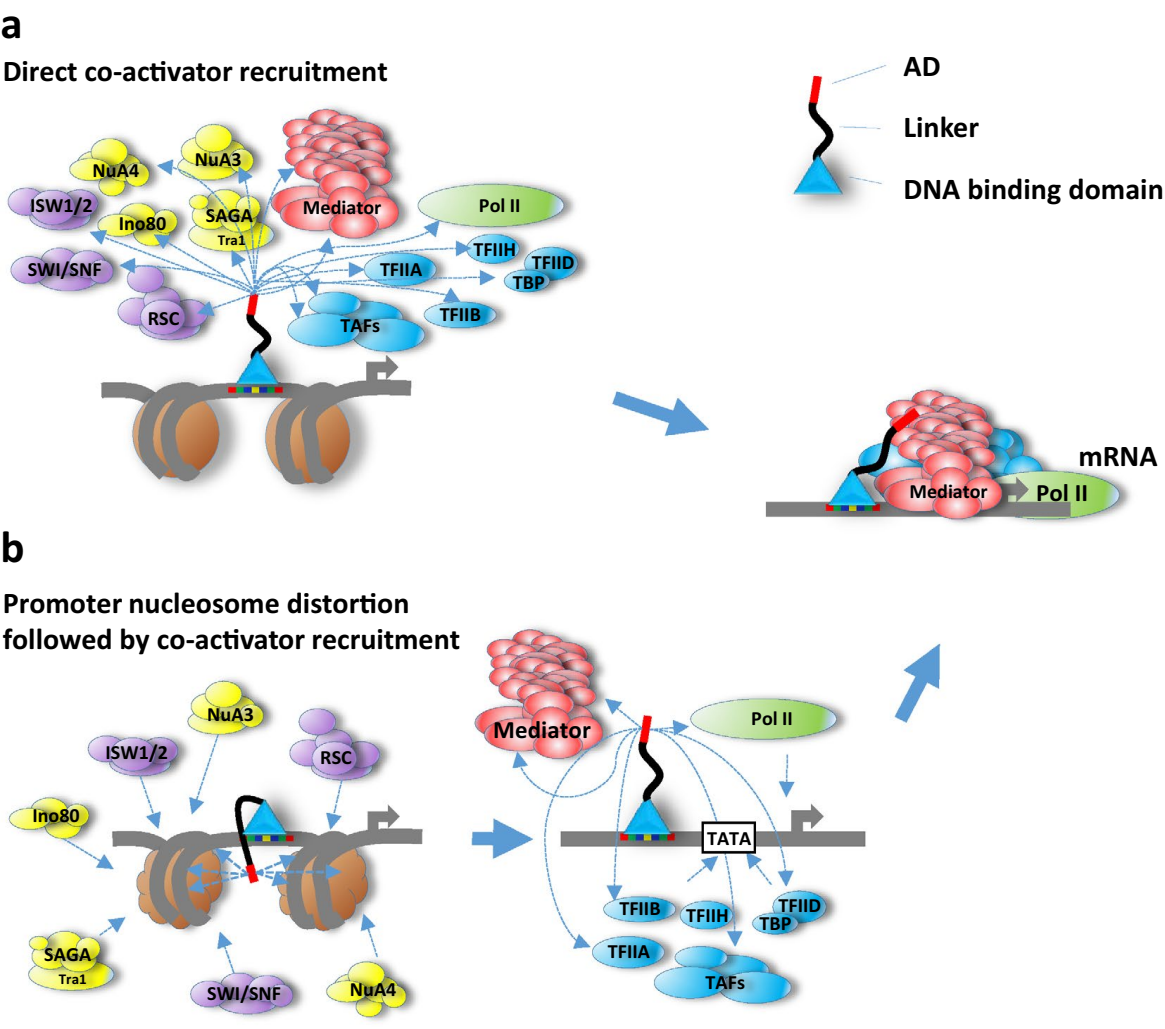
Traditional model of direct co-activator recruitment and an alternative model postulating initial promoter nucleosome distortion followed by co-activators and basal factors recruitment and assembly of transcription initiation complex. **a** The direct-recruitment model suggests numerous physical interaction of AD with multiple co-activators and transcription initiation complex components, thus bringing them to the promoter. **b** In the promoter nucleosome-distortion model, the AD modifies promoter nucleosome structure triggering an action of enzymatic activities, thus further affecting nucleosome structure, which leads to the nucleosome translocation and promoter opening. After this stage, the AD in cooperation with freely exposed DNA promoter elements recruits and stabilizes at the promoter the transcription initiation complex components

In an attempt to help with solving problems formulated above, we propose to consider additional mechanistic options that involve local interaction of ADs with promoter nucleosomes. This possibility naturally arises from the fact that a majority of ADs are negatively charged and hydrophobic, thus likely interacting with positively charged and hydrophobic targets. Another premise for our model is that general chromatin remodeling at gene promoters is a well-established and often required step leading to an activation of transcription. According to the AD–nucleosome interaction model, the otherwise unlikely and weakly interacting partners—ADs and nucleosomes—are brought into proximity by the DBD of the activator and interact only in the vicinity of the activator-binding site due to this facilitation by DBD. Interactions of ADs are likely possible at multiple points on nucleosomes and, while being of low specificity, are leading to a structural destabilization of promoter nucleosomes, and either to a translocation of nucleosomes from the promoter, or to an exposure for the chromatin remodelers and histone modifiers triggering additional chromatin-remodeling events. ADs, which remain at the nucleosome-free promoter anchored by DBD, later participate in direct recruitment and more importantly stabilization of transcription initiation complex. Thus, ADs may play a dual function of initially triggering nucleosome distortion and translocation, and later attracting and stabilizing transcription initiation complex (Fig. 1).

There are significant distinctions in the interaction of ADs (with interaction partners) in the direct-recruitment model and in the nucleosome-distortion model. For the recruitment model, the requirement is an establishment of a functional link by attraction of a specific enzymatic activity(s) to the promoter; thus, the more functionally productive interactions have to have higher affinity to specific targets interacting in a specific structural region of the target as it was demonstrated for TBP, Gal11, Tra1, and others [15, 87, 104, 105]. In contrast, for the nucleosome-distortion model, in order to avoid nucleosome stabilization at the promoter (as this outcome impedes transcription initiation), the interactions must be of low or very low affinity, occurring likely in multiple structural points of the nucleosome with these points competing with each other and preventing creation of a stable link and thus maximally destabilizing the nucleosome. Importantly, these interactions are transient, significantly facilitated by DBD of the activator, and thus possible only in the vicinity of the activator-binding site. The function of AD in the nucleosome-distortion model can be compared to the function of a mild DBD-directed detergent that partially dissolves promoter nucleosomes. The nucleosome-distortion model thus is highly compatible with the absence of a clear consensus sequence in ADs as well as with intrinsic structural disorder of ADs.

Invoking AD–nucleosome interaction model provides solutions to all of the questions raised above:

1. Low-affinity “fuzzy” interactions, instead of creating a conundrum of mutually disruptive and competitive recruitment events of co-activators [2], are actually an important and unavoidable requirement of the AD–nucleosome-distortion model.
2. Considering the requirement of low- or very low-specificity destabilizing interactions in multiple points of nucleosomes, it is clear why selection of functional ADs from random pools [26, 31, 32] has never been shown to produce a specific AD consensus sequence. The same reasoning can be applied to answering the question of why selection of native ADs during actual biological evolution led to such great variability and absence of a consensus sequence in ADs.
3. As a result of AD–promoter nucleosome interactions, the recruitment of distinct enzymatic activities to a specific gene promoter can be dictated, at least in some situations, by the chromatin architecture of a specific promoter and by a specific nucleosome distortion induced by a specific AD. An additional source of specificity could be the particular composition of promoter DNA elements, such as in TATA- and TATA-less promoters.
4. The intrinsically disordered structural nature of ADs is a feature compatible with and actually favorable for “fuzzy” interactions with different destabilizing points of nucleosomes leading to the subsequent recruitment of co-activators.
5. Finally, the retention of functionality of ADs during the transfer of the same sequence between very distant biological phyla is also easily explainable by extremely high evolutionary conservation of histones and nucleosome structure. In contrast, the evolutionary conservation of co-activator complexes is significantly lower. Consequently, for the direct-recruitment model, this would dictate a significant drop of the AD activation potential during transfer into an evolutionarily distant species.

The proposition of the nucleosome-distortion model can be supported by the following considerations. First, the abundance of histones in the cell is at least one to two orders of magnitude higher than the abundance of any interacting targets of ADs [106] described above, and nucleosomes are present at almost any gene promoter. Considering that histones are predominantly nuclear proteins, the ratio for histones is even greater and may exceed three orders of magnitude in the nucleus. Second, taking into account that the initial stage of AD–target interactions might be nonspecific charge scanning [15, 103], it is logical to compare the exposure of positive charges on the surface of nucleosome and frequently considered AD recruitment targets. While nucleosome (PDB ID 3AFA) has 134 solvent-exposed positively charged (lysine and arginine) amino acid residues, GAL11 (Med15) has 111 total positive charges (no structure available), and TBP (PDB ID 1CDW) has only 32. Considering that a nucleosome usually is positioned in the vicinity of the activator-binding site at the promoter, while the co-activator targets are not, the initial charge scanning by AD happens more likely on the nucleosome surface.

There are many ways the nucleosome structure can be disturbed; however, for nucleosome relocation, at least some of the histone–DNA contacts must first be broken. Considering that AD sequences typically can be reduced to eight or nine amino acids [21, 27] or even smaller functional blocks [13] and that the critical residues in ADs are negatively charged and highly hydrophobic (usually aromatic) [26, 27, 31, 32], the simplest way to distort the histone–DNA contacts would be by interaction of ADs with positively charged lysine or arginine residues of histones and aromatic bases of DNA [107], perhaps by intercalation. The average distance between the histone octamer surface amino groups of arginines or lysines to the closest DNA base (human nucleosome PDB ID 3afa) lies between four and 16 Å, with the average being between 10 and 12 Å. Considering that one amino acid in the extended conformation covers approximately 3.5 Å, these distance intervals are in good agreement with the typical AD length [21, 26, 27, 31, 32].

### Fundamental difficulties of detecting AD–nucleosome interactions

The in vivo possibility of AD–nucleosome interactions was demonstrated in early experiments. By modified yeast two-hybrid screening, it was shown that histone H3 is among possible targets of such transcriptional activators as human CTF1/NF1 [108] and yeast Hap1 [109]. It was demonstrated also that the histone-binding module of mouse HNF3 is important for function of this activator [110]. For the CTF1/NF1 activator, the histone H3 interacting module was pinpointed to a 14-amino-acid stretch, and it was demonstrated that the splice variants that lack this sequence lose the transcription activation potential [111, 112]. Importantly, the histone H3-binding peptide is able to function as a synthetic AD while fused to DBD of either GAL4 or as a part of HSF1 [31].

These early experiments demonstrating the possibility of AD–nucleosome interactions, in comparison with the demonstration of direct recruitment of co-activators, are certainly not numerous, and critics may ask why. The answer might lie in the nature of the AD–nucleosomal contacts postulated by the AD nucleosome-distortion model. Considering this model, the challenges of demonstration of AD–nucleosome interactions become obvious:

1. These interactions are transient and of very low specificity, possible only with facilitation by the activator’s DBD and only to nucleosomes in close proximity of the activator-binding site. This extremely low-specificity requirement directly originates from experimental data showing high randomness of AD sequences (1–2 % of random pools), which are able to substitute the native ADs [26, 31, 32]. One percentage of a random pool as well as short size of typically 10–15 amino acids with only few critical ones suggests interactions of extremely low specificity at the level of noise. These kinds of interactions in classical biochemical experiments (pull-downs, cross-linking, etc.) are considered as nonessential and are typically ignored or eliminated by experimental procedures as background or noise.
2. AD interactions with nucleosomes likely occur at multiple low-affinity points which compete with each other. Contrary to the proposed model, any high-affinity interactions would lead to nucleosome stabilization and repression of transcription. The idea that ADs interact with nucleosomes at multiple competing points is also compatible with the multiplicity of AD sequences that can function in identical molecular contexts [26, 31, 32]. The fact that a majority of ADs are intrinsically disordered also supports this supposition. However, the detection of these transient, low-specificity interactions occurring in multiple points of the target is challenging even for such sensitive and tolerant to high Kd method as NMR.
3. The distorting interactions of AD with nucleosomes are likely less strong and specific than with co-activators and transcriptional PIC components. Thus, in typical biochemical experiments, they are likely to be ignored, leaving interactions with co-activators and PIC as the dominant ones and thus more obvious for mechanistic interpretations of AD function.
4. The difficulties with explaining the mechanisms of AD function [3, 8, 15, 16], which persist despite enormous experimental efforts for more than three decades, can well be rooted in conceptual fixation on relatively strong physically connecting and specific co-activator recruitment interactions, as well as in the methodological ease of detection and experimental manipulation of these interactions. The long-standing experimental and conceptual challenges with mechanisms of AD function are typical for the whole newly emerging field of intrinsically disordered protein regions. This field became prominent only recently, after the realization that a majority of protein regions in higher eukaryotes contain parts that are intrinsically disordered, or at least not locked into rigid structures; however, this disorder and randomness are more prominent in vivo and often required for proper function [41]. Designing of experiments directly demonstrating and proving functionality of low-specificity interactions at the noise level is a formidable challenge for classical biochemistry and might require fundamental reevaluation of methodological approaches.

## Conclusions

The function of ADs has been a fundamental enigma of the transcription regulation field for more than 30 years. One of the reasons we face this problem arguably is due to the traditional approach, which considers vital protein–protein interactions in terms of the key-and-lock concept or an approximation of it, looking for specific or semi-specific interactions between AD and possible target(s), and utilizing classical biochemistry methods designed to detect these specific interactions and discard noisy background. Our review and analysis is an attempt to get away from this stereotype, and to switch to a reality of intrinsically disordered regions functioning at an extremely low-specificity level. The model of transcription activation based on promoter nucleosome distortion by AD requires these extremely low-affinity noise-level interactions and is an addition to the classical direct-recruitment model (Fig. 1). Our proposed model resolves the AD sequence specificity problem, as well as associated problems with AD target selection. The extremely low specificity of AD–target interactions at the biochemical noise level, akin to the action of a mild detergent, underscores the fundamental challenges associated with experimental proof of the model and requires reevaluation of classical methodological approaches.

## Abbreviations

AD: transcription activation domain
DBD: DNA-binding domain
TAFs: transcription-associated factors
HSF: heat shock factor
TBP: TATA-binding protein

## References

[1] Ptashne M, Gann A (1997). Transcriptional activation by recruitment. Nature.

[2] Keung AJ, Bashor CJ, Kiriakov S, Collins JJ, Khalil AS (2014). Using targeted chromatin regulators to engineer combinatorial and spatial transcriptional regulation. Cell.

[3] Hahn S, Young ET (2011). Transcriptional regulation in Saccharomyces cerevisiae: transcription factor regulation and function, mechanisms of initiation, and roles of activators and coactivators. Genetics.

[4] Mapp AK, Ansari AZ, Ptashne M, Dervan PB (2000). Activation of gene expression by small molecule transcription factors. Proc Natl Acad Sci USA.

[5] Garvie CW, Wolberger C (2001). Recognition of specific DNA sequences. Mol Cell.

[6] Luscombe NM, Austin SE, Berman HM, Thornton JM. An overview of the structures of protein-DNA complexes. Genome Biol. 2000; 1(1):REVIEWS001. 10.1186/gb-2000-1-1-reviews001 PMC138832 11104519

[7] Hossain MA, Barrow JJ, Shen Y, Haq MI, Bungert J (2015). Artificial zinc finger DNA binding domains: versatile tools for genome engineering and modulation of gene expression. J Cell Biochem.

[8] Mapp AK, Ansari AZ (2007). A TAD further: exogenous control of gene activation. ACS Chem Biol.

[9] Rodriguez-Martinez JA, Peterson-Kaufman KJ, Ansari AZ (2010). Small-molecule regulators that mimic transcription factors. Biochim Biophys Acta.

[10] Eguchi A, Lee GO, Wan F, Erwin GS, Ansari AZ (2014). Controlling gene networks and cell fate with precision-targeted DNA-binding proteins and small-molecule-based genome readers. Biochem J.

[11] Ansari AZ, Mapp AK (2002). Modular design of artificial transcription factors. Curr Opin Chem Biol.

[12] Dervan PB (2001). Molecular recognition of DNA by small molecules. Bioorg Med Chem.

[13] Minter AR, Brennan BB, Mapp AK (2004). A small molecule transcriptional activation domain. J Am Chem Soc.

[14] Rowe SP, Casey RJ, Brennan BB, Buhrlage SJ, Mapp AK (2007). Transcriptional up-regulation in cells mediated by a small molecule. J Am Chem Soc.

[15] Warfield L, Tuttle LM, Pacheco D, Klevit RE, Hahn S (2014). A sequence-specific transcription activator motif and powerful synthetic variants that bind Mediator using a fuzzy protein interface. Proc Natl Acad Sci USA.

[16] Bhaumik SR, Green MR (2001). SAGA is an essential in vivo target of the yeast acidic activator Gal4p. Genes Dev.

[17] Titz B, Thomas S, Rajagopala SV, Chiba T, Ito T, Uetz P (2006). Transcriptional activators in yeast. Nucleic Acids Res.

[18] Beerli RR, Segal DJ, Dreier B, Barbas CF (1998). Toward controlling gene expression at will: specific regulation of the erbB-2/HER-2 promoter by using polydactyl zinc finger proteins constructed from modular building blocks. Proc Natl Acad Sci USA.

[19] Escher D, Bodmer-Glavas M, Barberis A, Schaffner W (2000). Conservation of glutamine-rich transactivation function between yeast and humans. Mol Cell Biol.

[20] Yanagisawa S (2001). The transcriptional activation domain of the plant-specific Dof1 factor functions in plant, animal, and yeast cells. Plant Cell Physiol.

[21] Piskacek S, Gregor M, Nemethova M, Grabner M, Kovarik P, Piskacek M (2007). Nine-amino-acid transactivation domain: establishment and prediction utilities. Genomics.

[22] Ma J, Przibilla E, Hu J, Bogorad L, Ptashne M (1988). Yeast activators stimulate plant gene expression. Nature.

[23] Liu J, Perumal NB, Oldfield CJ, Su EW, Uversky VN, Dunker AK (2006). Intrinsic disorder in transcription factors. Biochemistry.

[24] Minezaki Y, Homma K, Kinjo AR, Nishikawa K (2006). Human transcription factors contain a high fraction of intrinsically disordered regions essential for transcriptional regulation. J Mol Biol.

[25] Mitchell PJ, Tjian R (1989). Transcriptional regulation in mammalian cells by sequence-specific DNA binding proteins. Science.

[26] Ma J, Ptashne M (1987). A new class of yeast transcriptional activators. Cell.

[27] Lu X, Ansari AZ, Ptashne M (2000). An artificial transcriptional activating region with unusual properties. Proc Natl Acad Sci USA.

[28] Regier JL, Shen F, Triezenberg SJ (1993). Pattern of aromatic and hydrophobic amino acids critical for one of two subdomains of the VP16 transcriptional activator. Proc Natl Acad Sci USA.

[29] Cress WD, Triezenberg SJ (1991). Critical structural elements of the VP16 transcriptional activation domain. Science.

[30] Drysdale CM, Duenas E, Jackson BM, Reusser U, Braus GH, Hinnebusch AG (1995). The transcriptional activator GCN4 contains multiple activation domains that are critically dependent on hydrophobic amino acids. Mol Cell Biol.

[31] Erkine AM, Gross DS (2003). Dynamic chromatin alterations triggered by natural and synthetic activation domains. J Biol Chem.

[32] Abedi M, Caponigro G, Shen J, Hansen S, Sandrock T, Kamb A (2001). Transcriptional transactivation by selected short random peptides attached to lexA-GFP fusion proteins. BMC Mol Biol.

[33] Gerber HP, Seipel K, Georgiev O, Hofferer M, Hug M, Rusconi S, Schaffner W (1994). Transcriptional activation modulated by homopolymeric glutamine and proline stretches. Science.

[34] Blair WS, Bogerd HP, Madore SJ, Cullen BR (1994). Mutational analysis of the transcription activation domain of RelA: identification of a highly synergistic minimal acidic activation module. Mol Cell Biol.

[35] Wu Y, Reece RJ, Ptashne M (1996). Quantitation of putative activator-target affinities predicts transcriptional activating potentials. EMBO J.

[36] Buhrlage SJ, Bates CA, Rowe SP, Minter AR, Brennan BB, Majmudar CY, Wemmer DE, Al-Hashimi H, Mapp AK (2009). Amphipathic small molecules mimic the binding mode and function of endogenous transcription factors. ACS Chem Biol.

[37] Saha S, Ansari AZ, Jarrell KA, Ptashne M, Jarell KA (2003). RNA sequences that work as transcriptional activating regions. Nucleic Acids Res.

[38] Nyanguile O, Uesugi M, Austin DJ, Verdine GL (1997). A nonnatural transcriptional coactivator. Proc Natl Acad Sci USA.

[39] Tompa P (2012). Intrinsically disordered proteins: a 10-year recap. Trends Biochem Sci.

[40] Oldfield CJ, Dunker AK (2014). Intrinsically disordered proteins and intrinsically disordered protein regions. Annu Rev Biochem.

[41] Uversky VN (2015). The multifaceted roles of intrinsic disorder in protein complexes. FEBS Lett.

[42] Giniger E, Ptashne M (1987). Transcription in yeast activated by a putative amphipathic alpha helix linked to a DNA binding unit. Nature.

[43] Kussie PH, Gorina S, Marechal V, Elenbaas B, Moreau J, Levine AJ, Pavletich NP (1996). Structure of the MDM2 oncoprotein bound to the p53 tumor suppressor transactivation domain. Science.

[44] Radhakrishnan I, Perez-Alvarado GC, Parker D, Dyson HJ, Montminy MR, Wright PE (1997). Solution structure of the KIX domain of CBP bound to the transactivation domain of CREB: a model for activator:coactivator interactions. Cell.

[45] Jonker HR, Wechselberger RW, Boelens R, Folkers GE, Kaptein R (2005). Structural properties of the promiscuous VP16 activation domain. Biochemistry.

[46] Uesugi M, Nyanguile O, Lu H, Levine AJ, Verdine GL (1997). Induced alpha helix in the VP16 activation domain upon binding to a human TAF. Science.

[47] Lee CW, Martinez-Yamout MA, Dyson HJ, Wright PE (2010). Structure of the p53 transactivation domain in complex with the nuclear receptor coactivator binding domain of CREB binding protein. Biochemistry.

[48] Brent R, Ptashne M (1985). A eukaryotic transcriptional activator bearing the DNA specificity of a prokaryotic repressor. Cell.

[49] Horikoshi M, Hai T, Lin YS, Green MR, Roeder RG (1988). Transcription factor ATF interacts with the TATA factor to facilitate establishment of a preinitiation complex. Cell.

[50] Stringer KF, Ingles CJ, Greenblatt J (1990). Direct and selective binding of an acidic transcriptional activation domain to the TATA-box factor TFIID. Nature.

[51] Hermann S, Berndt KD, Wright AP (2001). How transcriptional activators bind target proteins. J Biol Chem.

[52] Capella M, Re DA, Arce AL, Chan RL (2014). Plant homeodomain-leucine zipper I transcription factors exhibit different functional AHA motifs that selectively interact with TBP or/and TFIIB. Plant Cell Rep.

[53] Khan SH, Ling J, Kumar R (2011). TBP binding-induced folding of the glucocorticoid receptor AF1 domain facilitates its interaction with steroid receptor coactivator-1. PLoS ONE.

[54] Lin YS, Ha I, Maldonado E, Reinberg D, Green MR (1991). Binding of general transcription factor TFIIB to an acidic activating region. Nature.

[55] Choy B, Green MR (1993). Eukaryotic activators function during multiple steps of preinitiation complex assembly. Nature.

[56] Xiao H, Pearson A, Coulombe B, Truant R, Zhang S, Regier JL, Triezenberg SJ, Reinberg D, Flores O, Ingles CJ (1994). Binding of basal transcription factor TFIIH to the acidic activation domains of VP16 and p53. Mol Cell Biol.

[57] Chabot PR, Raiola L, Lussier-Price M, Morse T, Arseneault G, Archambault J, Omichinski JG (2014). Structural and functional characterization of a complex between the acidic transactivation domain of EBNA2 and the Tfb1/p62 subunit of TFIIH. PLoS Pathog.

[58] Stargell LA, Struhl K (1995). The TBP-TFIIA interaction in the response to acidic activators in vivo. Science.

[59] Ozer J, Bolden AH, Lieberman PM (1996). Transcription factor IIA mutations show activator-specific defects and reveal a IIA function distinct from stimulation of TBP-DNA binding. J Biol Chem.

[60] Tan Q, Linask KL, Ebright RH, Woychik NA (2000). Activation mutants in yeast RNA polymerase II subunit RPB3 provide evidence for a structurally conserved surface required for activation in eukaryotes and bacteria. Genes Dev.

[61] Goodrich JA, Tjian R (1994). TBP-TAF complexes: selectivity factors for eukaryotic transcription. Curr Opin Cell Biol.

[62] Walker SS, Reese JC, Apone LM, Green MR (1996). Transcription activation in cells lacking TAFIIS. Nature.

[63] Moqtaderi Z, Bai Y, Poon D, Weil PA, Struhl K (1996). TBP-associated factors are not generally required for transcriptional activation in yeast. Nature.

[64] Kim YJ, Bjorklund S, Li Y, Sayre MH, Kornberg RD (1994). A multiprotein mediator of transcriptional activation and its interaction with the C-terminal repeat domain of RNA polymerase II. Cell.

[65] Koleske AJ, Young RA (1994). An RNA polymerase II holoenzyme responsive to activators. Nature.

[66] Aguilar X, Blomberg J, Brannstrom K, Olofsson A, Schleucher J, Bjorklund S (2014). Interaction studies of the human and Arabidopsis thaliana Med25-ACID proteins with the herpes simplex virus VP16- and plant-specific Dreb2a transcription factors. PLoS ONE.

[67] Biddick R, Young ET (2005). Yeast mediator and its role in transcriptional regulation. CR Biol.

[68] Koh SS, Ansari AZ, Ptashne M, Young RA (1998). An activator target in the RNA polymerase II holoenzyme. Mol Cell.

[69] Myers LC, Gustafsson CM, Hayashibara KC, Brown PO, Kornberg RD (1999). Mediator protein mutations that selectively abolish activated transcription. Proc Natl Acad Sci USA.

[70] Jeong CJ, Yang SH, Xie Y, Zhang L, Johnston SA, Kodadek T (2001). Evidence that Gal11 protein is a target of the Gal4 activation domain in the mediator. Biochemistry.

[71] Ansari AZ, Koh SS, Zaman Z, Bongards C, Lehming N, Young RA, Ptashne M (2002). Transcriptional activating regions target a cyclin-dependent kinase. Proc Natl Acad Sci USA.

[72] Qiu H, Hu C, Yoon S, Natarajan K, Swanson MJ, Hinnebusch AG (2004). An array of coactivators is required for optimal recruitment of TATA binding protein and RNA polymerase II by promoter-bound Gcn4p. Mol Cell Biol.

[73] Rachez C, Lemon BD, Suldan Z, Bromleigh V, Gamble M, Naar AM, Erdjument-Bromage H, Tempst P, Freedman LP (1999). Ligand-dependent transcription activation by nuclear receptors requires the DRIP complex. Nature.

[74] Malik S, Roeder RG (2000). Transcriptional regulation through Mediator-like coactivators in yeast and metazoan cells. Trends Biochem Sci.

[75] Malik S, Roeder RG (2005). Dynamic regulation of pol II transcription by the mammalian Mediator complex. Trends Biochem Sci.

[76] Ito M, Yuan CX, Okano HJ, Darnell RB, Roeder RG (2000). Involvement of the TRAP220 component of the TRAP/SMCC coactivator complex in embryonic development and thyroid hormone action. Mol Cell.

[77] Reeves WM, Hahn S (2003). Activator-independent functions of the yeast mediator sin4 complex in preinitiation complex formation and transcription reinitiation. Mol Cell Biol.

[78] Svaren J, Schmitz J, Horz W (1994). The transactivation domain of Pho4 is required for nucleosome disruption at the PHO5 promoter. EMBO J.

[79] Stafford GA, Morse RH (1997). Chromatin remodeling by transcriptional activation domains in a yeast episome. J Biol Chem.

[80] Moreira JM, Holmberg S (1998). Nucleosome structure of the yeast CHA1 promoter: analysis of activation-dependent chromatin remodeling of an RNA-polymerase-II-transcribed gene in TBP and RNA pol II mutants defective in vivo in response to acidic activators. EMBO J.

[81] Di Mauro E, Kendrew SG, Caserta M (2000). Two distinct nucleosome alterations characterize chromatin remodeling at the *Saccharomyces cerevisiae* ADH2 promoter. J Biol Chem.

[82] Sullivan EK, Weirich CS, Guyon JR, Sif S, Kingston RE (2001). Transcriptional activation domains of human heat shock factor 1 recruit human SWI/SNF. Mol Cell Biol.

[83] Workman JL, Kingston RE (1998). Alteration of nucleosome structure as a mechanism of transcriptional regulation. Annu Rev Biochem.

[84] Fry CJ, Peterson CL (2001). Chromatin remodeling enzymes: who’s on first?. Curr Biol.

[85] Hassan AH, Neely KE, Vignali M, Reese JC, Workman JL (2001). Promoter targeting of chromatin-modifying complexes. Front Biosci.

[86] Knutson BA, Hahn S (2011). Domains of Tra1 important for activator recruitment and transcription coactivator functions of SAGA and NuA4 complexes. Mol Cell Biol.

[87] Brown CE, Howe L, Sousa K, Alley SC, Carrozza MJ, Tan S, Workman JL (2001). Recruitment of HAT complexes by direct activator interactions with the ATM-related Tra1 subunit. Science.

[88] Bhaumik SR, Raha T, Aiello DP, Green MR (2004). In vivo target of a transcriptional activator revealed by fluorescence resonance energy transfer. Genes Dev.

[89] Majmudar CY, Labut AE, Mapp AK (2009). Tra1 as a screening target for transcriptional activation domain discovery. Bioorg Med Chem Lett.

[90] Barlev NA, Candau R, Wang L, Darpino P, Silverman N, Berger SL (1995). Characterization of physical interactions of the putative transcriptional adaptor, ADA2, with acidic activation domains and TATA- binding protein. J Biol Chem.

[91] Thut CJ, Chen JL, Klemm R, Tjian R (1995). p53 transcriptional activation mediated by coactivators TAFII40 and TAFII60. Science.

[92] Henriksson A, Almlof T, Ford J, McEwan IJ, Gustafsson JA, Wright AP (1997). Role of the Ada adaptor complex in gene activation by the glucocorticoid receptor. Mol Cell Biol.

[93] Neely KE, Hassan AH, Wallberg AE, Steger DJ, Cairns BR, Wright AP, Workman JL (1999). Activation domain-mediated targeting of the SWI/SNF complex to promoters stimulates transcription from nucleosome arrays. Mol Cell.

[94] Neely KE, Hassan AH, Brown CE, Howe L, Workman JL (2002). Transcription activator interactions with multiple SWI/SNF subunits. Mol Cell Biol.

[95] Prochasson P, Neely KE, Hassan AH, Li B, Workman JL (2003). Targeting activity is required for SWI/SNF function in vivo and is accomplished through two partially redundant activator-interaction domains. Mol Cell.

[96] Kundu TK, Palhan VB, Wang Z, An W, Cole PA, Roeder RG (2000). Activator-dependent transcription from chromatin in vitro involving targeted histone acetylation by p300. Mol Cell.

[97] Lau OD, Kundu TK, Soccio RE, Ait-Si-Ali S, Khalil EM, Vassilev A, Wolffe AP, Nakatani Y, Roeder RG, Cole PA (2000). HATs off: selective synthetic inhibitors of the histone acetyltransferases p300 and PCAF. Mol Cell.

[98] Mukherjee SP, Behar M, Birnbaum HA, Hoffmann A, Wright PE, Ghosh G (2013). Analysis of the RelA:CBP/p300 interaction reveals its involvement in NF-kappaB-driven transcription. PLoS Biol.

[99] Cosma MP, Tanaka T, Nasmyth K (1999). Ordered recruitment of transcription and chromatin remodeling factors to a cell cycle- and developmentally regulated promoter. Cell.

[100] Cosma MP, Panizza S, Nasmyth K (2001). Cdk1 triggers association of RNA polymerase to cell cycle promoters only after recruitment of the mediator by SBF. Mol Cell.

[101] Sigler PB (1988). Transcriptional activation. Acid blobs and negative noodles. Nature.

[102] Ansari AZ, Reece RJ, Ptashne M (1998). A transcriptional activating region with two contrasting modes of protein interaction. Proc Natl Acad Sci USA.

[103] Ferreira ME, Hermann S, Prochasson P, Workman JL, Berndt KD, Wright AP (2005). Mechanism of transcription factor recruitment by acidic activators. J Biol Chem.

[104] Maeda R, Suzuki H, Tanaka Y, Tamura TA (2014). Interaction between transactivation domain of p53 and middle part of TBP-like protein (TLP) is involved in TLP-stimulated and p53-activated transcription from the p21 upstream promoter. PLoS ONE.

[105] Yuan CX, Gurley WB (2000). Potential targets for HSF1 within the preinitiation complex. Cell Stress Chaperones.

[106] Wang M, Weiss M, Simonovic M, Haertinger G, Schrimpf SP, Hengartner MO, von Mering C (2012). PaxDb, a database of protein abundance averages across all three domains of life. Mol Cell Proteomics.

[107] Waters ML (2002). Aromatic interactions in model systems. Curr Opin Chem Biol.

[108] Alevizopoulos A, Dusserre Y, Tsai-Pflugfelder M, von der Weid T, Wahli W, Mermod N (1995). A proline-rich TGF-beta-responsive transcriptional activator interacts with histone H3. Genes Dev.

[109] Ha N, Hellauer K, Turcotte B (2000). Fusions with histone H3 result in highly specific alteration of gene expression. Nucleic Acids Res.

[110] Cirillo LA, Lin FR, Cuesta I, Friedman D, Jarnik M, Zaret KS (2002). Opening of compacted chromatin by early developmental transcription factors HNF3 (FoxA) and GATA-4. Mol Cell.

[111] Altmann H, Wendler W, Winnacker EL (1994). Transcriptional activation by CTF proteins is mediated by a bipartite low-proline domain. Proc Natl Acad Sci USA.

[112] Wenzelides S, Altmann H, Wendler W, Winnacker EL (1996). CTF5—a new transcriptional activator of the NFI/CTF family. Nucleic Acids Res.

